# Implied object direction from eye location enhances animacy ratings but not detection of chasing behavior

**DOI:** 10.1038/s41598-025-08681-0

**Published:** 2025-07-01

**Authors:** Takahiro Kawabe

**Affiliations:** NTT Communication Science Laboratories, 3-1, Morinosato Wakamiya, Atsugi, 243-0198 Kanagawa Japan

**Keywords:** Animacy, Intention, Eye location, Object shape, Motion, Wolfpack effect, Psychology, Human behaviour

## Abstract

Understanding how humans perceive animacy in dynamic visual stimuli is fundamental to elucidating the mechanisms underlying visual social cognition. While both object geometry and eye-like features are known to independently influence animacy impressions, their interactive effects remain insufficiently explored. This study investigates how the combination of object geometry and the location of eye-like patterns modulates the perception of animacy in non-living moving objects. In Experiment 1, we manipulated the pointing direction of triangular objects and the location of eye-like features (near the vertex, near the edge, or absent), and found that animacy impressions were enhanced when the direction of motion aligned with the gaze-implied object direction, irrespective of the object’s geometrical shape. Experiment 2 examined whether this effect generalizes to an objective task, in which participants identify a target triangle chasing a green disk among distractors. Although the gaze-implied object direction did not significantly influence detection sensitivity, the spatial location of the eye pattern within the triangular shape continued to weakly modulate participants’ performance. These findings suggest that cues related to eye location contribute to subjective animacy at multiple stages of cognitive processing, with their impact varying depending on task demands and perceptual context.

**Supplementary Information:**

The online version contains supplementary material available at 10.1038/s41598-025-08681-0.

## Introduction

The human cognitive system excels at extracting rich and meaningful information from dynamic visual signals^1^. These signals are particularly crucial for determining whether an encountered entity is alive^2,3^. Among such signals, image motion^4^ and dynamic shape^5,6^ serve as robust cues for biological motion perception, which is closely linked to animacy perception^7^.

In the context of image motion, the trajectory pattern of a moving object plays a pivotal role^8–11^. For example, changes in the direction and speed of a non-living object’s motion significantly influence animacy impressions^11^. Additionally, two-dimensional 1/f noise trajectories have been shown to evoke animacy perception^12,13^ and even trigger predatory behavior in Medaka fish^14^. Such trajectories may inherently contain kinematic features that effectively induce animacy impressions^15^.

The geometry of a moving object also modulates animacy impressions. A key factor is the alignment between an object’s motion trajectory and its orientation^11^, with misalignments reducing animacy impressions. Beyond human observers, Rosa-Salva et al.^16,17^ demonstrated that visually naive domestic chicks (Gallus gallus domesticus) are sensitive to the alignment between an object’s motion axis and its principal shape axis, implying that analogous animacy cues operate across phylogenetically distant species. Using dart-shaped items, Gao and his colleagues^18,19^ demonstrated the “Wolfpack effect,” where the ability to detect social relationships (e.g., chaser/chased dynamics) between objects was impaired when the dart shapes of the objects pointed toward a task-irrelevant target. Interestingly, a similar effect occurred when an eye pattern (two red dots resembling eyes) served as a directional cue instead of the dart shape. As social relationships inherently relate to animacy, the Wolfpack effect suggests that the directional properties of moving objects contribute to animacy impressions.

Eyes are particularly powerful cues for animacy perception. Eye patterns on non-living objects enhance animacy impressions^20–22^. In human faces, eyes are among the strongest animacy cues^23^, although exceptions exist^24^.　The existence of eyes in the robot’s face facilitates the anthropomorphic attribution of the robot’s behavior^25^.

It is known that when eye-like patterns are added to moving inanimate objects, the position of these “eyes” can influence impressions of animacy. A previous study^26^ demonstrated this by placing eye-like patterns on the top surface of a cube-shaped robot whose movement was controlled by a Perlin noise trajectory. Observers rated the animacy of the robot, and the results showed that animacy impressions were enhanced when the robot’s direction of motion aligned with the gaze-implied object direction, which refers to the perceived facing direction inferred from eye location within an object.

In these ways, while the effects of object geometrical shape and eye patterns on animacy impressions have been studied individually, their interaction remains unclear. Investigating this interaction could provide deeper insights into the heuristic image features the cognitive system uses to judge animacy. Furthermore, it could inform the design of animacy cues in images, animations, and robotic appearances. Animals typically have eyes on the side of their body that faces their direction of movement. Such an anteroposterior organization of an agent may influence how infants encode the direction of agency. For instance, Hernik et al. empirically measured the anticipatory waiting time of 12-month-old human infants observing an agent approaching a stationary box and demonstrated that this bodily organization significantly modulated the infants’ anticipatory waiting behavior. Upon the evidence, we hypothesized that animacy impressions of non-living objects would be enhanced if an eye pattern appeared on the side facing the direction of motion, regardless of the object’s geometrical shape.

This study conducted two experiments to explore the interaction between object’s geometrical shapes and eye locations in forming animacy impressions of non-living moving objects. Experiment 1 examined how animacy impressions were influenced by two factors: the direction of a triangular object and the location of its eyes. Prior studies indicate that an object’s pointedness influences its perceived motion direction^27^ and memory for its disappearance position^28^. Therefore, we used a triangular object with explicit pointedness as the stimulus. Participants rated two types of animacy impressions: “moving like a live animal” and “intentionality.” Prior research^29,30^ suggests these two impressions follow distinct patterns depending on stimulus parameters. Thus, we analyzed how eye location and motion direction influenced these impressions.

Experiment 2 investigated whether the effects of the gaze-implied object direction, which were observed in Experiment 1, would persist in a more objective task setting. The task was modeled after the paradigm used to study the Wolfpack effect^19^, and involved displays containing a moving green disk and several moving triangular objects. Participants were asked to detect a target triangle that appeared to be chasing the green disk.

Drawing on findings from both experiments, we discuss how eye location and object’s geometrical shapes interact to form the impressions of animacy in non-living moving objects, and how these cues may contribute to animacy impressions at multiple stages of visual cognition.

## Experiment 1

### Methods

#### Participants

A total of 187 individuals participated in this experiment. In the session where participants rated their impression of a live animal, 47 women (mean age = 48.7 years, SD = 6.94) and 48 men (mean age = 50.41 years, SD = 7.50) took part. In the session where participants rated their impression of intentionality, 43 women (mean age = 46.9 years, SD = 10.29) and 49 men (mean age = 47.6 years, SD = 8.08) participated. Each session was designed to include 94 participants, as determined by a prior power analysis using Morepower 6.0. This calculation was based on assumptions of a power of 0.8, an effect size (eta squared^31^, *η*^2^) of 0.02, a significance level (p) of 0.05, and an MSE of 1. *η*^2^ is a measure of effect size that represents the proportion of variance in a dependent variable explained by an independent variable in ANOVA. The final number of participants closely matched the target. All participants were Japanese nationals and were recruited online via a crowdsourcing research company based in Japan. While they were compensated for their participation, the specific amount was determined based on the company’s undisclosed criteria. The company selected only individuals capable of participating using a personal computer with a computer mouse. Participants were naive to the specific purpose of the experiments. Ethical approval for this study was obtained from the Ethics Committee of NTT Communication Science Laboratories (Approval number: R06-009). The experiments adhered to the principles outlined in the 2013 Declaration of Helsinki. Written informed consent was obtained from all participants prior to the study.

#### Apparatus

The experiment was conducted online, requiring participants to use their own personal computers to complete the task. Smartphones and tablet devices were excluded because the experimental program was only compatible with personal computers.

#### Stimuli

**Fig. 1:**
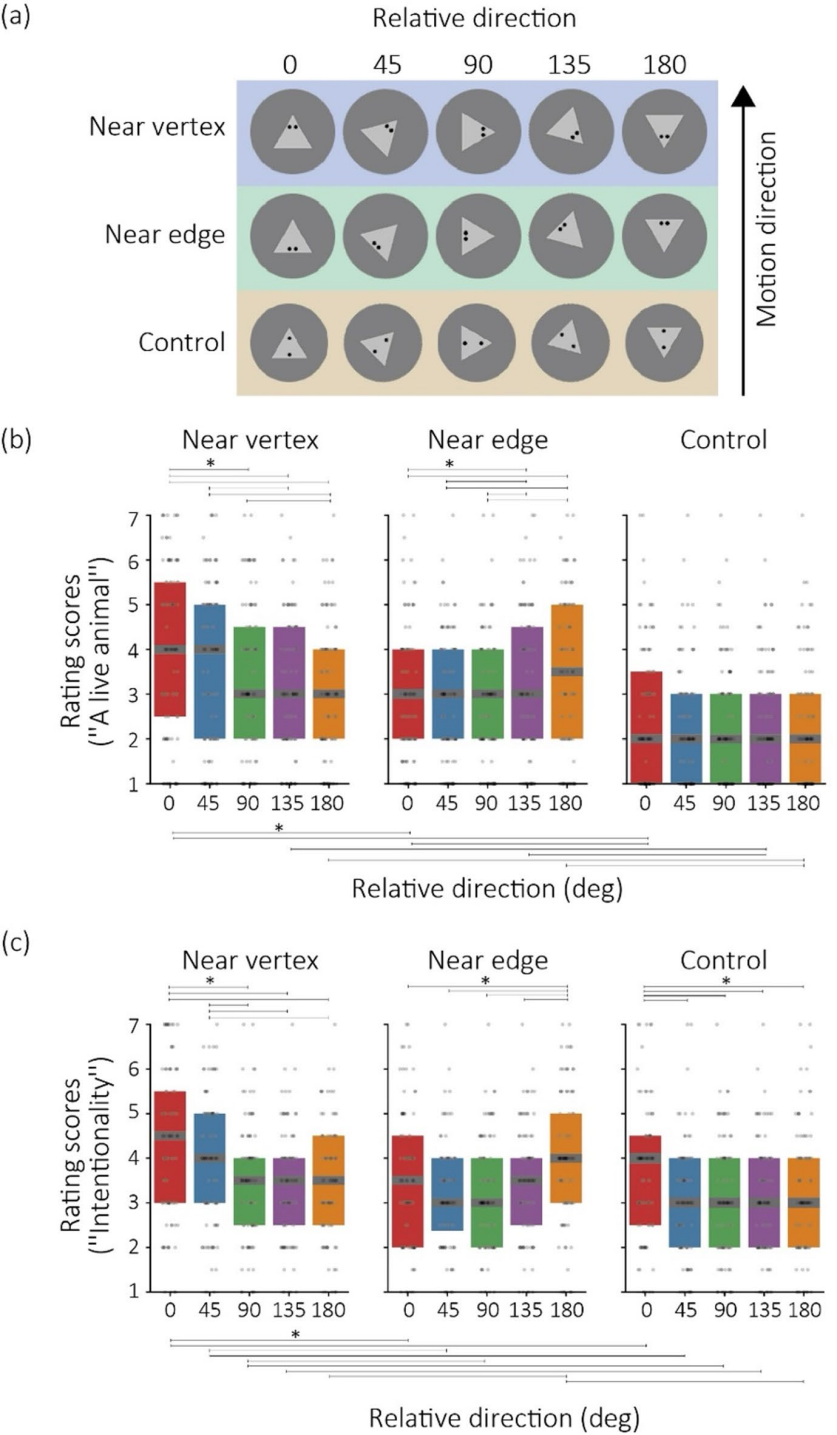
(**a**) Stimulus conditions used in Experiment 1. The experiment manipulated two factors: three types of eye locations and the relative direction between the motion direction and the pointing direction of the triangular object. (**b**, **c**) Box plots of rating scores for (**b**) the impression of a live animal and (**c**) the impression of intentionality. The horizontal thick lines within the boxes represent the median scores for each condition, while the top and bottom edges of the boxes indicate the 75th and 25th percentiles of the rating scores, respectively. Lines above and below the graph panels indicate significant comparisons among the experimental conditions. Asterisks have been placed above these lines to denote significance (*p* < 0.05).

The stimuli consisted of video clips (see Supplementary movies 1) depicting a triangular object in motion (Fig. 1a). The object was light gray (RGB: [192, 192, 192]), with each side of the triangle measuring 42 pixels. The clips were 800 pixels wide and 600 pixels high, with a gray background (RGB: [128, 128, 128]). In our online study, only the pixel dimensions of the clips were controlled. The object started moving from the center of the frame. The motion of the object was updated on each frame, which was refreshed every 33.3 milliseconds. Both the horizontal and vertical vectors of the object’s trajectory were determined by independent Perlin noise functions. Specifically, a Perlin noise pattern was generated for each trial to manipulate the motion trajectory over 10 s (300 frames). This noise was regenerated randomly for each clip, ensuring the object’s trajectory varied between the clips. The noise intensity was normalized to range between − 1.5 and 1.5, and the resulting values were used to define the trajectory vectors in units of pixels. Average speed of the object among the clips was 46.79 and standard deviation of the clip was 5.367. Two experimental factors were controlled in this study: Eye Location: The position of the eye pattern within the triangle and Relative Direction: The relationship between the motion direction of the object and the orientation of its pointed apex (i.e., object direction). Details of these factors are described as follows.

1. Eye location. We tested three conditions for eye location: near vertex, near edge, and Control. In the near vertex condition, two black dots (RGB: [32, 32, 32]), resembling an eye pattern, were positioned near one of the corners of the triangle. In the near edge condition, the two black dots were placed along one side of the triangle. In the control condition, the two black dots were arranged along the symmetrical axis of the triangle. The distance between the dots was greater than in Type 1 and Type 2, creating a less pronounced impression of the eye pattern.
2. Relative direction. We manipulated the relative direction between the motion direction and the pointed direction of the triangular object. The 0-degree condition was defined as the case where the motion direction aligned with the vertex of the triangle. The 180-degree condition occurred when the motion direction was opposite to the pointedness of the triangle. Additionally, intermediate conditions of 45 deg, 90 deg (orthogonal), and 135 deg were also tested. In total, five levels of relative direction were assessed.

For each combination of eye location and relative direction, four clips with unique motion trajectories were generated. Consequently, a total of 60 clips were tested, corresponding to 3 types of eye locations, 5 levels of relative direction, and 4 unique variations per combination.

#### Procedure

In each trial, participants were presented with a video clip. After viewing the stimulus, one group of participants was asked to rate the intensity with which they perceived the impression of a live animal (i.e., the “live animal impression”) in the moving objects using a 7-point scale. The other group was asked to rate the extent to which they perceived the moving object as intentionally moving (i.e., the “intention impression”). Instructions were provided in Japanese via text displayed above the stimuli (See Supplementary Fig. 1). Participants provided their ratings by selecting a score via radio buttons displayed on the screen. The next trial began 500 milliseconds after the participant submitted their rating. Each participant completed 60 trials, comprising a combination of 3 eye location types × 5 levels of the relative direction between motion and object directions × 4 variations. The task took approximately 10–15 min to complete. The order of trials was pseudo-randomized to ensure variation across participants.

#### Statistics

Since the rating scores were not normally distributed, applying parametric tests directly to the raw rating scores was deemed inappropriate. Therefore, following the methods of previous studies^32,33^, the scores were transformed into ranked data. Using the ranked data, we performed a two-way repeated-measures analysis of variance (ANOVA), with eye location and relative direction as within-participant factors. The p-values in the post hoc tests were adjusted using the Bonferroni method. Although *η*^2^ was used for the a priori power analysis conducted in MorePower 6.0, partial eta-squared^34^ (*η*^2^_*p*_) will be reported in the results, which is the conventional measure for repeated-measures ANOVA. One advantage of *η*^2^_*p*_ is that it provides an estimate of the effect size independent of other factors in the model, allowing for a more isolated interpretation of each factor’s contribution to the variance. This property is particularly useful in multifactorial or within-subject designs. Note that *η*^2^ and *η*^2^_p_ are conceptually distinct and not directly comparable.

### Results

Figure 1b and c present box and scatter plots showing the rating scores for the groups reporting impressions of “a live animal” and “intentionality,” respectively.

#### Live animal impression

The results of the ANOVA, including multiple comparison tests for the significant simple effects, are provided in Supplementary Data 1. The main effect of the eye location was significant [*F*(2, 188) = 52.218, *p* < 0.0001, *η*^*2*^_*p*_ = 0.357]. The main effect of the relative direction was also significant [*F*(4, 376) = 15.108, *p* < 0.0001, *η*^*2*^_*p*_ = 0.138]. The interaction of the two factors was also significant [*F*(8, 752) = 20.300, *p* < 0.0001, *η*^*2*^_*p*_ = 0.177]. Based on the significant interaction, we computed the simple effects.

In the near vertex condition, the simple main effect of the relative direction was significant [*F*(4, 376) = 24.425, *p* < 0.0001, *η*^*2*^_*p*_ = 0.206]. Multiple comparison tests showed that in the near vertex condition, the score in the 0 deg condition was significantly higher than that in the 90, 135, 180 deg conditions (*p* < 0.05). The score in the 45 deg condition was significantly higher than that in the 135 and 180 deg conditions (*p* < 0.05). The score in the 90 deg condition was significantly higher than that in the 180 deg condition (*p* < 0.05). In the near edge condition, the simple main effect of the relative direction was significant [*F*(4, 376) = 12.899, *p* < 0.0001, *η*^*2*^_*p*_ = 0.120]. Multiple comparison tests showed that the scores in the 0, 45, and 90 deg conditions are significantly lower than those in the 135 and 180 deg conditions (*p* <. 05).

The simple main effect of the eye location was significant when the relative directions were 0 deg [*F*(2, 188) = 48.998, *p* < 0.0001, *η*^*2*^_*p*_ = 0.342], 45 deg [*F*(2, 188) = 41.529, *p* < 0.0001, *η*^*2*^_*p*_ = 0.306], 90 deg [*F*(2, 188) = 34.613, *p* < 0.0001, *η*^*2*^_*p*_ = 0.269], 135 deg [*F*(2, 188) = 25.52, *p* < 0.0001, *η*^*2*^_*p*_ = 0.213], and 180 deg [*F*(2, 188) = 32.858, *p* < 0.0001, *η*^*2*^_*p*_ = 0.259]. When the relative directions were 0°, 45°, and 90°, each eye location condition differed significantly from the others (*p* < 0.05). When the relative directions were 135 and 180 deg, the control condition was significantly lower than the other two conditions (*p* <  .05).

#### Intention impression

The results of the ANOVA, including multiple comparison tests for the significant simple effects, are provided in Supplementary Data 2. The main effect of the eye location was significant [*F*(2, 182) = 24.286, *p* < 0.0001, *η*^*2*^_*p*_ = 0.210]. The main effect of the relative direction was also significant [*F*(4, 364) = 15.305, *p* < 0.0001, *η*^*2*^_*p*_ = 0.143]. The interaction of the two factors was also significant [*F*(8, 728) = 14.054, *p* < 0.0001, *η*^*2*^_*p*_ = 0.133]. Based on the significant interaction, we computed the simple effects. The results of multiple comparison tests for the significant simple main effect are described as follows.

In the near vertex condition, the simple main effect of the relative direction was significant [*F*(4, 364) = 19.776, *p* < 0.0001, *η*^*2*^_*p*_ = 0.178]. Multiple comparison tests showed that the scores in the 0 and 45 deg conditions were significantly higher than those in the 90, 135, and 180 deg conditions (*p* < 0.05). In the near edge condition, the simple main effect of the relative direction was significant [*F*(4, 364) = 13.926, *p* < 0.0001, *η*^*2*^_*p*_ = 0.132]. Multiple comparison tests showed that the score in the 180 deg condition was significantly higher than that in the 0, 45, 90, and 135 deg conditions (*p* < 0.05). In the control condition, the simple main effect of the relative direction was significant [*F*(4, 364) = 7.425, *p* < 0.0001, *η*^*2*^_*p*_ = 0.07]. The score in the 0 deg condition was significantly different from that in the 45, 90, 135, and 180 deg conditions (*p* < 0.05).

The simple main effect of the eye location was significant when the relative directions were 0 deg [*F*(2, 182) = 19.568, *p* < 0.0001, *η*^*2*^_*p*_ = 0.176], 45 deg [*F*(2, 182) = 28.147, *p* < 0.0001, *η*^*2*^_*p*_ = 0.236], 90 deg [*F*(2, 182) = 8.068, *p* = 0.0004, *η*^*2*^_*p*_ = 0.08], 135 deg [*F*(2, 182) = 4.085, *p* = 0.0183, *η*^*2*^_*p*_ = 0.042], and 180 deg [*F*(2, 182) = 19.006, *p* < 0.0001, *η*^*2*^_*p*_ = 0.172]. In the 0, 45, and 90 deg condition, the score in the near vertex condition was significantly higher than that in the near edge and control conditions (*p* < 0.05). In the 135 deg condition, the score in the near edge condition was significantly higher than the score in the control condition (*p* < 0.05). In the 180 deg condition, the score in the near edge condition was significantly higher than that in the near vertex and control conditions (*p* < 0.05).

### Discussion

The results in general indicated, animacy impressions of a moving object were primarily influenced by the gaze-implied object direction. Where the eyes were located near the vertex of a triangular object, animacy impressions increased as the relative difference between the triangle’s pointing direction and its moving direction decreased. In contrast, when the eyes were positioned near the edge of the triangle, animacy impressions were strongest when the relative direction was 180 deg—that is, when the triangle’s pointing direction was completely opposite to its moving direction. These findings suggest that regardless of the object geometry, the gaze-implied object direction is a key factor influencing the animacy impressions we tested.

On the other hand, note that no significant difference was observed between the near vertex and near edge condition when the relative direction was 180 deg while there was a significant difference between them when the relative direction was 0 deg. In the near vertex condition, where the eyes are located near the vertex of the triangular object, the configuration likely establishes a scenario where the pointedness of the triangular object aligns with the gaze-implied object direction in the 0 deg condition. Conversely, in the near edge condition, where the eyes are positioned farther from the vertex, the configuration likely establishes a scenario where the pointedness of the object is inconsistent with the gaze-implied object direction in the 180 deg condition. The set of results suggest that the cognitive system relies on the consistency between the gaze-implied object direction and the pointedness of the object as a cue to determine animacy impressions.

In the control condition, the triangle’s pointing direction did not influence the impression of a live animal but did affect the impression of intentionality. The spatial arrangement of the two dots in the control condition aligns with the bisectors of the triangle’s angles. This arrangement of the dots might have served as a cue for conveying the orientation of the object. Consequently, under the condition where the relative direction was 0 deg, the dot pattern, functioning as an orientation cue, and the object’s pointedness may have interacted synergistically, enhancing the “intentionality” impression.

Although the results clearly demonstrated a significant effect of the gaze-implied object direction on animacy impressions, it remains unclear whether this effect extends to more objective task settings. Recent research has suggested that the influence of orientation on animacy ratings may be attributed to higher-level reasoning rather than low-level perceptual processes^35^. This raises the question of whether the effect of the gaze-implied object direction on animacy impressions similarly reflects cognitive inference rather than perceptual mechanisms. To address this, we conducted the following experiment.

## Experiment 2

### Purpose

The aim of this experiment was to examine whether the influence of the gaze-implied object direction on animacy impressions would also manifest in an objective task. Previous studies have employed paradigms in which participants were asked to detect a triangular item chasing a green disk among a set of moving distractor items. The perception of chasing behavior is regarded as a fundamental aspect of social interaction among living agents and, as such, is closely linked to animacy perception^36^. Prior findings have shown that when distractor items are oriented toward a task-irrelevant target, the detection of chasing behavior becomes more difficult^19^. Building on this paradigm, we developed a task in which participants judged whether a moving triangular object was chasing a moving green disk, presented among other triangular distractors moving randomly. If the influence of the gaze-implied object direction on animacy evaluations extends to this kind of objective task, then detection performance should deteriorate when the gaze-implied object direction is oriented toward the green disk, regardless of the physical pointedness of the triangle.

### Methods

#### Participants

Twenty-two individuals (mean age = 31.68 years, SD = 11.20, 11 females) participated in this experiment. The sample size was determined using Morepower 6.0 to ensure that a 2 × 3 repeated-measures ANOVA would detect an effect size (*η*^2^ of 0.2, with an alpha level of 0.05 and a statistical power (1 − *β*) of 0.80, which indicated that a minimum of 22 participants was required. Ethical approval for this study was obtained from the Ethics Committee of NTT Communication Science Laboratories (Approval number: R06-013). The experiments adhered to the principles outlined in the 2013 Declaration of Helsinki. Written informed consent was obtained from all participants prior to the study.

#### Apparatus

Stimuli were presented on a 120 Hz LCD monitor (Display++, Cambridge Research Systems Inc., USA) with a luminance range of 0 to 144 cd/m^2^. A Mac Pro computer (Apple Inc., USA) was used to control stimulus presentation and data collection. The experimental scripts were written by using PsychoPy^37^ .

#### Stimuli

**Fig. 2:**
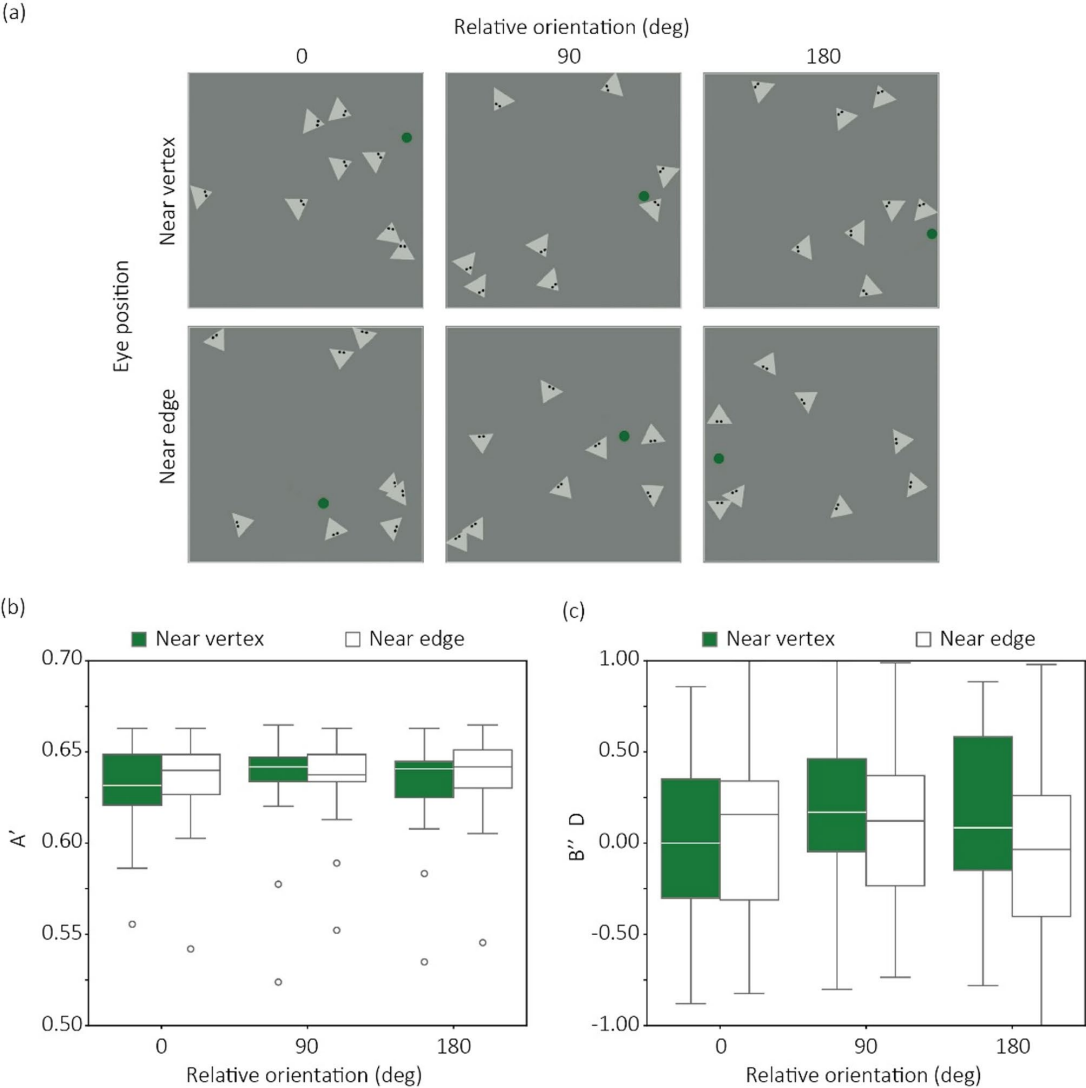
(**a**) Example snapshots from stimulus clips for each experimental condition. (**b**) A′ as a function of relative orientation for each eye location. (**c**) B″D as a function of relative orientation for each eye location.

As illustrated in Fig. 2a, we manipulated two factors: eye location (near vertex vs. near edge) and relative direction (0°, 90°, and 180°). In each display, a green disk [RGB (0, 192, 0)] with a diameter of 0.54° of visual angle moved randomly within a square area measuring 14° × 14° against a neutral gray background (72.2 cd/m^2^). Eight triangular objects, each measuring 0.67° per side and presented at a luminance of 101 cd/m^2^, were also displayed within the same area, with their eye locations and relative orientations systematically varied. When the relative direction was 0°, all triangles pointed directly toward the green disk. When it was 180°, the flat edge of each triangle faced the disk. In the 90° condition, the triangles were oriented orthogonally, facing neither directly toward nor away from the disk. Both the disk and triangular objects changed their direction by 90° whenever it reached the boundary of the area. In half of the trials, one of the triangular objects chased the green disk by following its trajectory with a temporal delay of 1.5 s. The speed of both the green disk and the triangular objects was kept constant at 6.5°/sec. The presentation duration of each clip was 3 s. See Supplementary movies 2 for the appearance of stimuli.

#### Procedure

Participants used a chin-and-head rest to stabilize their visual field. They viewed the stimuli from a distance of 57.3 cm from an LCD display. Each trial was initiated by pressing the spacebar. Five hundred milliseconds after the keypress, a stimulus clip featuring the green disk and the triangular objects began. The clip lasted for 3 s, after which it disappeared from the screen. Participants were then asked to report whether a triangle had been chasing the green disk. Each participant completed 720 trials, consisting of a 2 (eye location: near vertex or near edge) × 3 (relative orientation: 0°, 90°, or 180°) × 2 (target condition: present or absent) design, with 60 repetitions per condition. The trials were divided into 10 blocks, and trial order within each block was randomized. Between blocks, participants were given short breaks lasting approximately 5–10 min.

#### Statistics

We performed a two-way repeated-measures analysis of variance (ANOVA), with eye location and relative direction as within-participant factors.

### Results

Figure 2b and c shows the sensitivity A’^38^ and the response bias B’’D^39^ for detecting the target triangle chasing the green disk. For each index, we conducted the ANOVA as described above. For A’, the main effect of the eye location was significant [*F*(1,21) = 7.88, *p* = 0.010, *η*^*2*^_*p*_ = 0.27]. The main effect of relative orientation was not significant [*F*(2,42) = 0.957, *η*^*2*^_*p*_ = 0.04, *p* = .39]. Interaction between the two factors was not significant [*F*(2,42) = 0.805, *η*^*2*^_*p*_ = 0.04, *p* = .0.453]. For B’’D, the main effect of the eye location was significant [*F*(1,21) = 4.357, *η*^*2*^_*p*_ = 0.17, *p* = 0.049]. The main effect of relative orientation was not significant [*F*(2,42) = 0.881, *η*^*2*^_*p*_ = 0.04, *p* = .42]. Interaction between the two factors was not significant [*F*(2,42) = 1.548, *η*^*2*^ = 0.06, *p* = .0.22].

### Discussion

The results showed that the effect of the gaze-implied object direction was not observed in the objective task. If this effect had generalized to the objective task, a significant interaction between eye location and relative orientation would have been expected—specifically, reduced performance in the near-vertex condition with a relative orientation of 0°, or in the near-edge condition with a relative orientation of 180°. Thus, consistent with previous findings suggesting that the effect of orientation on animacy ratings can be attributed to cognitive inference^35^, the present results indicate that the influence of the gaze-implied object direction on animacy impressions similarly arises from higher-level reasoning about the relationship between eye location and object orientation (i.e., the pointedness of the triangle).

However, we did find a significant main effect of eye location on detection sensitivity (A’) and bias (B’’D). Specifically, performance deteriorated when the eyes were located near the vertex compared to when they were near the edge, irrespective of relative orientation. Moreover, the response was more conservative in the near vertex condition than in the near edge condition. These results suggest that eye location and object geometry (specifically, pointedness) interact at perceptual and decision levels and influence certain stages of processing involved in detecting chasing–chased relationships. In contrast, the interaction between the gaze-implied object direction and object geometry appears to emerge only at the level of cognitive inference.

## General discussion

The first experiment demonstrated that the gaze-implied object direction significantly influenced animacy evaluations. In contrast, the second experiment revealed that this effect did not emerge in the objective task. Instead, a significant main effect of eye location itself was observed.

The results of this study suggest that animacy impressions are shaped by multi-stage cognitive processing. The gaze-implied object direction did not influence performance in the objective task, indicating that the effect of implied direction and its interaction with object geometry (i.e., triangle pointedness) likely arises from higher-level cognitive inference. This interpretation aligns with previous findings, as the established effect of geometry on animacy ratings^11^ has also been attributed to cognitive-level inference^35^. To further investigate this issue, it would be valuable to conduct the Blindfold test^35^ to assess whether the effect of the gaze-implied object direction can be elicited even in the absence of actual visual stimuli, based solely on verbal descriptions of the stimuli.

In contrast, Experiment 2 revealed a significant effect of eye location on sensitivity and bias to detecting the chasing–chased relationship. These results suggest that eye location and triangle pointedness may be integrated at perceptual and decision levels, influencing the interpretation of the motion direction of triangular objects. When the eyes are positioned near the vertex, the static directional cue arising from the combination of eye placement and shape pointedness may be particularly strong. As a result, regardless of the relative orientation, this static cue could interfere with the perception of chasing behavior. Or, the static cue may cause more conservative responses when the eyes are positioned near the vertex. In contrast, when the eyes are located near the edge, the static directional cues may be weaker, thereby mitigating any decline in detection performance and bias. On the other hand, because the observed differences in A′ and B″D are minimal, it is necessary to interpret the effect of eye location on sensitivity and bias in detecting the chasing–chased relationship with caution. Furthermore, a limitation of this study is that the sample size was determined based on the assumption of a large effect size, which resulted in a relatively small sample size. Consequently, it is possible that weaker effects could not be detected.

The present study suggests that animacy may manifest differently at the perceptual level and the level of cognitive inference. Previous research has shown that some phenomena previously assumed to be processed at the perceptual level are, in fact, better explained in terms of cognitive inference^40^. Consistent with this, our findings support the conclusion that the effect of the gaze-implied object direction on animacy should be attributed to cognitive inference. On the other hand, the fact that the effect of eye location interfered with the detection of a chasing target suggests that this effect may also arise at the perceptual level. Thus, the results indicate that eye location, as a cue to animacy, may exert different influences at the perceptual and cognitive levels. That said, given the relatively small effect size, eye location may play only a minor role in animacy perception.

The interpretation aligns with predictive coding accounts of perception, which posit that sensory processing is shaped by top-down expectations and inferences^41–43^. From this perspective, the effect of eye position may not arise solely from bottom-up visual input, but rather from cognitive inferences that shape perceptual experience. Furthermore, our findings are consistent with dual-process models^44^, suggesting that the animacy attribution and gaze interpretation observed in our study may involve higher-level processes.

The present study focused on examining the impact of the gaze-implied object direction on animacy impressions. However, other perceptual and cognitive cues associated with directional information in living beings may also play a role. For example, spatial patterns resembling fur could serve as perceptual cues for directionality, and the overall distribution of an object geometry may likewise provide information about its facing direction. Regarding the latter, a recent study^45^ has demonstrated that the global distribution of a complex object’s geometry can serve as a predictive oculomotor cue for the direction in which the object is expected to move. As described in the Introduction, visually naive domestic chicks (Gallus gallus domesticus) are sensitive to the parallel alignment between an object’s motion axis and its principal shape axis^16,17^, suggesting that certain forms of motion–shape integration may contribute to animacy perception without engaging higher-level cognitive reasoning. However, it remains unclear whether such shape-based cues interact with eye location to modulate the perception or impression of animacy in moving objects. Investigating how the global geometry of an object interacts with eye location and motion direction presents an intriguing avenue for future research—one that we have recently begun to explore.

It is also important to address the ecological validity of the stimuli used in the present study. In our stimuli, the moving object was a simple triangle—an uncommon geometry in everyday visual scenes. While the pointedness of the triangle may serve as a cue for animacy perception or reasoning, the effect of implied gaze direction might have been diminished due to the lack of the ecological validity in object geometry. In realistic contexts, gaze direction typically produces a strong attentional effect^46^, but our stimuli may not have captured this due to the abstract and unnatural geometry of the object. To enhance ecological validity in future studies, it may be necessary to use object geometries that more closely resemble real animals, such as fish or birds.

## Electronic supplementary material

Below is the link to the electronic supplementary material.

Supplementary Material 1

Supplementary Material 2

Supplementary Material 3

Supplementary Material 4

Supplementary Material 5

Supplementary Material 6

## Data Availability

The datasets used and/or analysed during the current study available from the corresponding author on reasonable request.
