# Supplementary material for: Implied object direction from eye location enhances animacy ratings but not detection of chasing behavior
Source: Sci Rep. 2025 Jul 1;15:20754. doi: 10.1038/s41598-025-08681-0 (PMC12214860; doi:10.1038/s41598-025-08681-0)
Supplement: Supplementary file 5 — Supplementary Material 5 [file 41598_2025_8681_MOESM5_ESM.pdf]

Supplementary Video 1. Example stimulus clips presented in Experiment 1.

Supplementary Video 2. Example stimulus clips presented in Experiment 2.
