# Supplementary material for: Implied object direction from eye location enhances animacy ratings but not detection of chasing behavior
Source: Sci Rep. 2025 Jul 1;15:20754. doi: 10.1038/s41598-025-08681-0 (PMC12214860; doi:10.1038/s41598-025-08681-0)
Supplement: Supplementary file 6 — Supplementary Material 6 [file 41598_2025_8681_MOESM6_ESM.pdf]

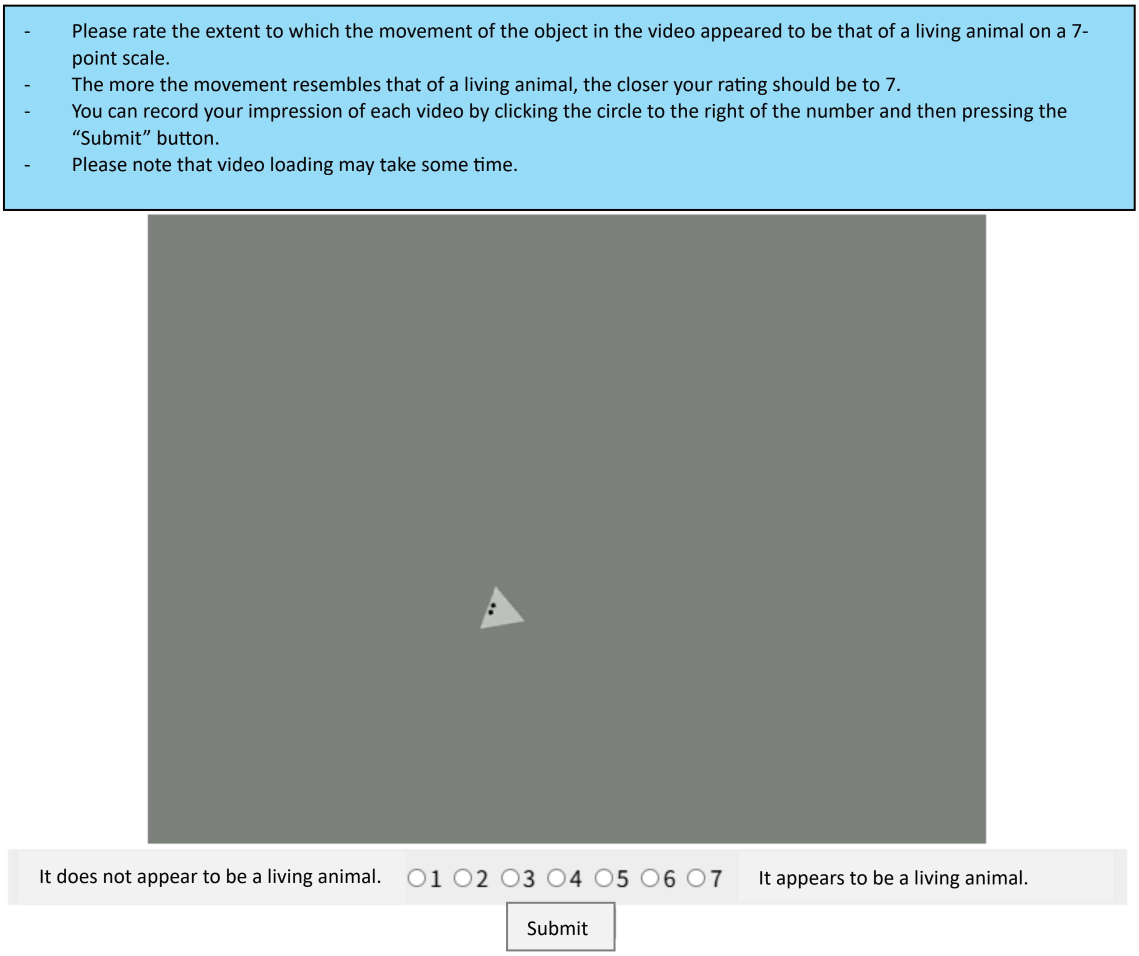


**Supplementary Figure 1.** A snapshot from a trial in Experiment 1. Instructions were originally presented in Japanese as text displayed above the stimuli. (In this figure, the English translation of the instructions is shown.)
